# Metabolomics Analysis of the Peels of Different Colored Citrus Fruits (*Citrus reticulata* cv. ‘Shatangju’) During the Maturation Period Based on UHPLC-QQQ-MS

**DOI:** 10.3390/molecules25020396

**Published:** 2020-01-17

**Authors:** Feiyan Wang, Yongjing Huang, Wen Wu, Congyi Zhu, Ruimin Zhang, Jiezhong Chen, Jiwu Zeng

**Affiliations:** 1 College of Horticulture, South China Agricultural University, Guangzhou 510642, China; wfei08@163.com; 2 Institute of Fruit Tree Research, Guangdong Academy of Agricultural Sciences, Key Laboratory of South Subtropical Fruit Biology and Genetic Resource Utilization & Guangdong Province Key Laboratory of Tropical and Subtropical Fruit Tree Research, Guangzhou 510640, China; yjhgzh@126.com (Y.H.); wuwen8@126.com (W.W.); zhucongyi@hotmail.com (C.Z.); xmingz@163.com (R.Z.)

**Keywords:** *Citrus reticulata* cv. ‘Shatangju’, Huanglongbing (HLB), metabolomics, PCA, OPLS-DA, UHPLC-QQQ-MS

## Abstract

Citrus is a globally consumed fruit with great popularity. Mandarin (*Citrus reticulata* cv. ‘Shatangju’) is a local variety, and its planting area and yield are the greatest regarding fruit tree planting in Guangdong Province, China. However, its resistance to Huanglongbing (HLB) is weak. After infection by HLB, the fruits cannot develop normally. In this study, four kinds of fruits were classified as HBG, XQG, ZQG, and DHG, according to the color of their peels. The metabolomes of the three abnormally colored groups (HBG, XQG, and ZQG) and the normally colored group (DHG) were compared using a UPLC-QQQ-MS-based metabolomics approach. In total, 913 metabolites were identified and classified into 23 different categories, including phenylpropanoids and flavonoids; among them, 215 (HBG, 177; XQG, 124; and ZQG, 62) metabolites showed differential accumulation in the three comparison groups (HBG/XQG/ZQG versus DHG). A total of 2 unique metabolites, *O*-caffeoyl maltotriose and myricetin were detected only in DHG samples. When comparing HBG with DHG, there were 109 decreased and 68 increased metabolites; comparing XQG with DHG, there were 88 decreased and 36 increased metabolites; comparing ZQG with DHG, 41 metabolites were decreased, and 21 metabolites were increased. Metabolic pathway enrichment analysis of these differential metabolites showed significant enrichment of the “phenylpropanoid biosynthesis” pathway in all comparison groups. The hierarchical cluster analysis of the differential metabolites of the four groups showed a clear grouping patterns. The relative contents of three phenylpropanoids, four flavonoids, two alkaloids, one anthocyanin, and two other metabolites were significantly different between each comparison group. This study might provide fundamental insight for the isolation and identification of functional compounds from the peels of citrus fruit infected with HLB and for in-depth research on the effect of HLB on the formation of fruits pigment and the development of HLB-resistant citrus varieties.

## 1. Introduction

Citrus is an important crop mainly applied in food industries for fresh juice production, and the peel is the main byproduct of its processing. *Citrus reticulata* cv. ‘Shatangju’ is one of the superior native citrus varieties in Guangdong Province, China. Its fruits are rich in polymethoxylated flavonoids (PMFs), vitamin C, folate, dietary fiber, and carotenoids and are very popular because of their nutrients and pigment [1,2,3]. In recent years, Huanglongbing (HLB), caused by *Candidatus* Liberibacter sp., has greatly affected the planting area and yield of ‘Shatangju’. The typical symptom of trees infected with HLB is that the affected leaves develop a pattern of yellow and green areas and arise asymmetrical on the two halves of the leaf [4,5]. Symptomatic fruit are small, lopsided, and when they mature and ripen, the stylar end remains green, such as sweet orange [4]. But most infected fruits of ‘Shatangju’ become “red nose” fruits [5]. This means that most fruits cannot develop normally and thus generate abnormal pigment and smaller fruits. Few fruits on an infected tree can develop normally into yellow and large fruits that are similar to the fruits of uninfected trees [6]. According to previous research, the pulp properties of impacted fruit are the main research focus, such as the sugar-acid ratio and flavor, and the peels have not been well investigated [6]. Previous studies have shown that metabolites are involved in many biological functions in the development of fruits, such as pigment, antifungal, and antioxidant properties [7,8,9].

The metabolism of plants is conventionally classified as producing primary and secondary metabolites. Primary metabolites are generally considered to be significant to fundamental plant life activities, including the metabolism of carbohydrates, lipids, and proteins. Secondary metabolites are mainly related to the synthesis of plant products and the formation of characteristic features, such as aroma and color [10,11,12]. The primary and secondary metabolites compose the complex metabolic network in plants. Metabolomics, as one of the post-genome era members, is the branch of science concerned with the quantitative understanding of the metabolite components of integrated living systems (organisms, tissues or cells) and their dynamic responses to changes in both endogenous and exogenous factors. Plant metabolomics can yield a large amount of qualitative and quantitative data on metabolites according to the organism-specific metabolic pathways, metabolic networks, and functions that the metabolites participate in; thus, biologically meaningful data can be retrieved [13]. Plant metabolomic studies have been of value for the identification of metabolites, regional differences, natural variations, and intraspecific and interspecific differences in citrus fruits [14,15], figs [16], and tomatoes [17]. Therefore, plant metabolomics can be a good tool to improve upon plant research.

Although some scientific studies have previously reported on the pigment, antifungal, and antioxidant composition of peels [18], little information is available on the use of metabolomics as a tool for analyzing the integral metabolites of citrus peels infected with HLB. In this study, four types of citrus fruits infected with HLB, including three abnormally colored fruits (HBG, XQG, and ZQG) and one normal colored fruit (DHG), were selected as experimental materials in order to have a better understanding of the differential metabolic profile of their peels and to clarify unique metabolites affected by Huanglongbing and associate with the pigment of citrus peels. Ultra-high-performance liquid chromatography coupled to triple quadrupole mass spectrometry (UHPLC-QQQ-MS) metabolomics approach was employed to analyze the types and relative contents of metabolites in the peels of different colored fruits infected with HLB. Metabolic pathway and hierarchical cluster analyses were also applied to identify the functional components associated with the pigment development of citrus peels. The results might facilitate a better understanding of metabolites between DHG peels and three other citrus peels and provide a theoretical basis for peels development of citrus fruits infected with HLB.

## 2. Results

### 2.1. Qualitative and Quantitative Analysis of Metabolites and Quality Control (QC) Analysis of Samples

**Figure 1:**
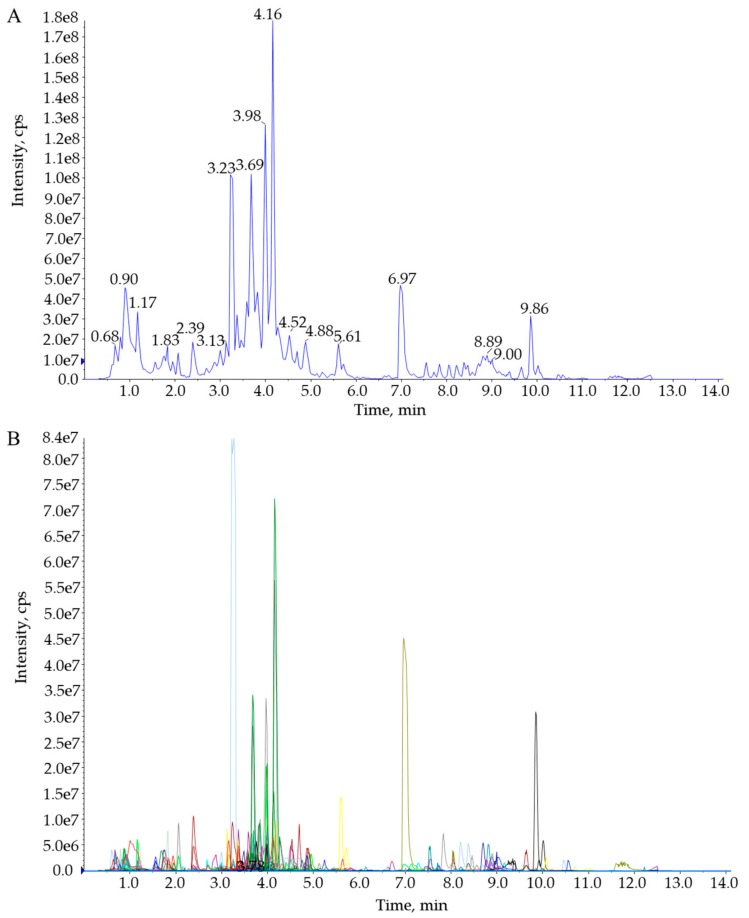
Total ion current of one quality control sample by mass spectrometry detection (**A**) and multi-peak detection plot of metabolites in the multiple reaction monitoring mode (**B**).

**Figure 2:**
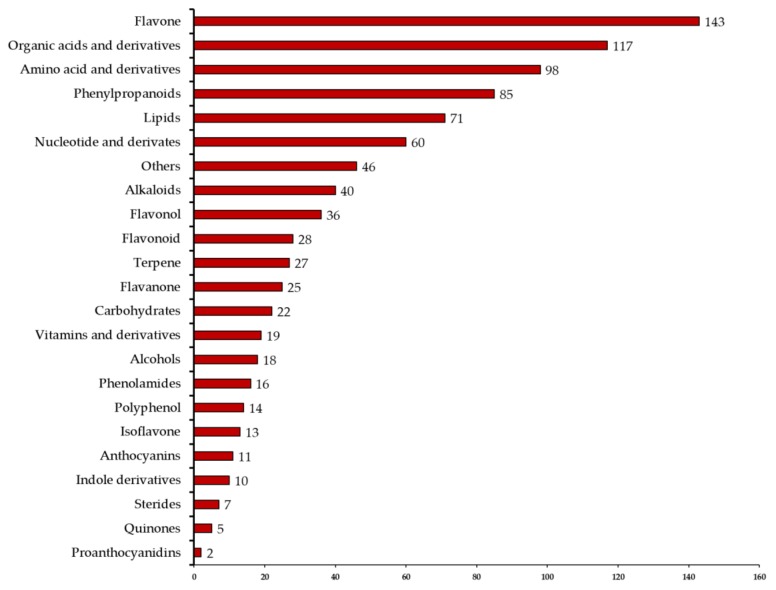
Number of different types of metabolites identified in all samples.

**Figure 3:**
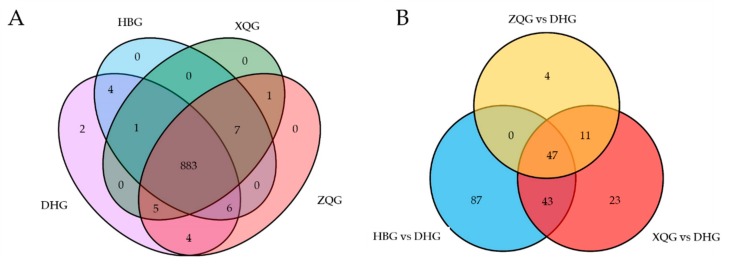
Venn diagrams of metabolites detected for each group (**A**) and differential metabolites for three comparison groups (**B**).

The total ion current (TIC) chromatograms of one quality control sample (QC) are shown in Figure 1A and show the summed intensity of all ions in the mass spectrum at different time points. The multipeak detection plot (XIC) of the metabolites in multiple reaction monitoring (MRM) mode is illustrated in Figure 1B, and the figure shows the ion current plot of multiple substances. The abscissa shows the retention time of the metabolites, and the ordinate shows the ion current intensity in counts per second (cps). The qualitative and quantitative mass spectrometry analyses were carried out on the metabolites in all samples based on the local metabolite database. In the MRM plot (Figure 1B), each colored mass spectral peak represents a detected metabolite, and the specific metabolite information, including serial numbers, peak integration values, and metabolite names, is listed in the Supplementary Information (Table S1). A total of 913 (DHG,905; HBG,901; XQG,897; ZQG,906; Figure 3A) metabolites were identified, including 143 flavones, 117 organic acids and their derivatives, 98 amino acids and their derivatives, 85 phenylpropanoids, 71 lipids, 60 nucleotides and their derivatives, 40 alkaloids, 36 flavonols, 28 flavonoids, 27 terpenes, 25 flavanones, 22 carbohydrates, 19 vitamins and their derivatives, 18 alcohols, 16 phenolamides, 14 polyphenols, 13 isoflavones, 11 anthocyanins, 10 indole derivatives, 7 sterides, 5 quinones, 2 proanthocyanidins, and 46 other metabolites (Figure 2, Figure 3A, Table S1). All metabolites of all samples were analyzed by ANOVA (Table S1), there were 593 metabolites that *p*-value was less than 0.05. As shown in the Figure 3A, most metabolites were detected and identified in all samples.

**Figure 4:**
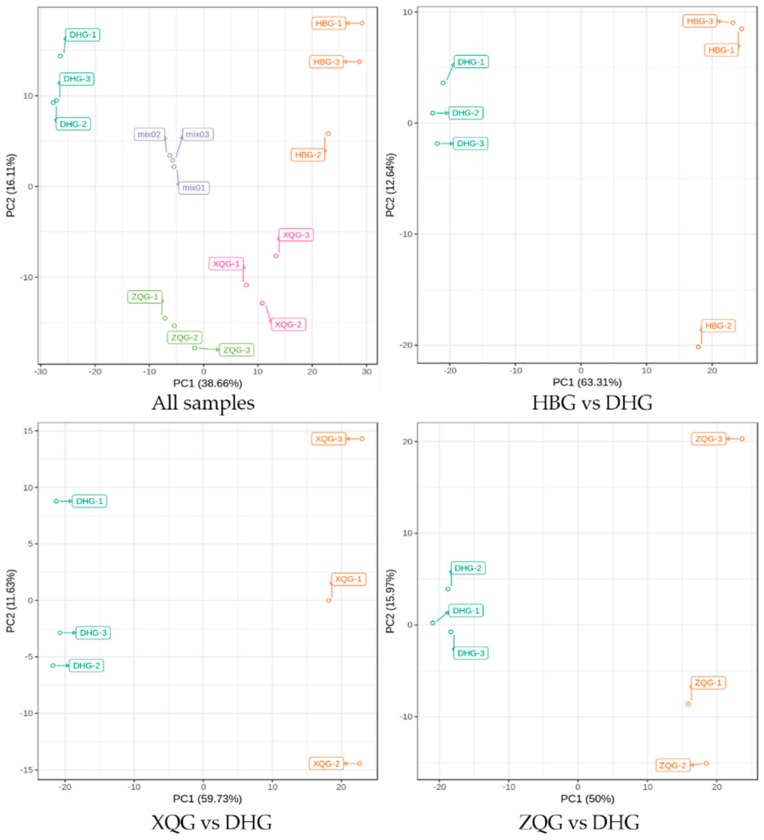
Principal component analysis (PCA) score plots for mass spectrum data of samples and quality control samples. The *x* axis represents the first principal component (PC1), and the *y* axis represents the second principal component (PC2).

The quality control samples (QC) were prepared by mixing extracts from all samples. Overlay analysis was applied to the TIC plots of different QC samples (mix) to evaluate the repeatability of the metabolite extractions and detections, namely, their technical repeatability. Figure S2 demonstrates the overlay of the TIC plots between the first and last QC samples. This result indicates a high degree of overlap in the TIC plots of metabolites, and the retention time (RT) and peak intensities were consistent between the QC samples. This signifies good signal stability in the detection of the same sample at different times. Moreover, all samples (including QC samples, mix) were subjected to principal component analysis (PCA) to investigate the overall metabolite differences among the different treatment groups and the intragroup variation in the metabolites. The PCA plot shows the scores of PC1 and PC2 (Figure 4) and suggests a large separation of trends among different treatment groups and little intragroup variation. Therefore, all results of the TIC evaluation and PCA suggested that all detected data in the present study demonstrate good repeatability and reliability.

### 2.2. PCA for the Three Treatment Groups (HBG, XQG, and ZQG) versus the Control Group

The abscissa and the ordinate of the PCA score plot for all samples, including the mixed samples, represent the scores of PC1 and PC2, respectively (Figure 4). The PCA plots show that the cumulative contribution rates reached 54.77%, 48.95%, 71.36%, and 65.97% in all samples groups, HBG, XQG, and ZQG comparison groups, respectively. The confidence intervals of all samples in Figure 4 were within 95%. In the PCA score plots, HBG, XQG, ZQG, and DHG were significantly separated, and the repeated samples of each group were flocked together (Figure 4). Therefore, the differences of metabolic components were subsistent among four groups samples and were significantly correlated with phenotypes of the fruits.

### 2.3. OPLS-DA for the Three Treatment Groups (HBG, XQG, and ZQG) versus the Control (DHG) Group

OPLS-DA can maximize the difference between groups and is used to screen differential metabolites. The OPLS-DA models could filter out orthogonal metabolite variables that were not related to categorical variables, and then nonorthogonal variables and orthogonal variables were analyzed to obtain reliable information about metabolite differences between the HBG/XQG/ZQG groups and the DHG group. The score plots generated from the intergroup comparison of each treatment group versus the control group in OPLS-DA are shown in Figure S3A. In the three plots, the abscissa (t1) all represent the predicted scores of PC1, and the ordinate (to1) all represent the scores of the orthogonal principal components. *Q*^2^ value greater than 0.9 indicates that the model is excellent. According to the results (HBG versus DHG, *R*^2^*Y* = 1, *Q*^2^ = 0.998; XQG versus DHG, *R*^2^*Y* = 1, *Q*^2^ = 0.999; ZQG versus DHG, *R*^2^*Y* = 1, *Q*^2^ = 0.992) in Figure S3A, all *Q*^2^ values in the three models are greater than 0.9 (Figure S3). These results demonstrate that all models are stable and reliable and could be applied to further screen for differential metabolites.

In the permutation test of OPLS-DA, the sequential order of the categorical variable *Y* was randomly changed many times (*n* = 200) for each of the three comparison groups. Therefore, on each occasion, one corresponding OPLS-DA model was established. The *R*^2^*Y* and *Q*^2^ values of the stochastic model were obtained. The original models *R*^2^*Y* and *Q*^2^ values were all close to 1 in the HBG, XQG, and ZQG comparison groups, indicating that the three established models were accurate descriptions of the real situation in each sample data and the distribution in each sample group would not be affected when new samples are added to the models (Figure S3B). The established original models of the three comparison groups can ideally explain the differences between each treatment group and the control group. The *R*^2^*Y*’ and *Q*^2^’ value of the stochastic models of the HBG, XQG, and ZQG comparison groups were less than the *R*^2^*Y* and *Q*^2^ values of the original models (Figure S3B). Therefore, these results indicate that the OPLS-DA models were stable and reproducible.

The OPLS-DA S-plots can intuitively represent the contribution rate of each metabolite to the group. As shown in Figure S3C, the abscissas (p [1]) of all plots represent the variable, and the ordinates (p(corr) [1]) of all plots represent the correlation between the samples. When a dot is farther away from the origin dot on the S-plots, the variable importance in project (VIP) value of the metabolite is larger, and thus the metabolite generates greater contribution to the classification (Figure S3C). The red dot represents that VIP value of metabolite is ≥1, and the green dot represents that the VIP value of metabolite is <1. Therefore, the S-plot shows the contribution of each metabolite for distinguishing between the four groups of samples.

### 2.4. Univariate Analysis and Screening and Venn and Volcano Plot Generation of Differential Metabolites for the Three Treatment Groups (HBG, XQG, and ZQG) versus the Control Group

**Figure 5:**
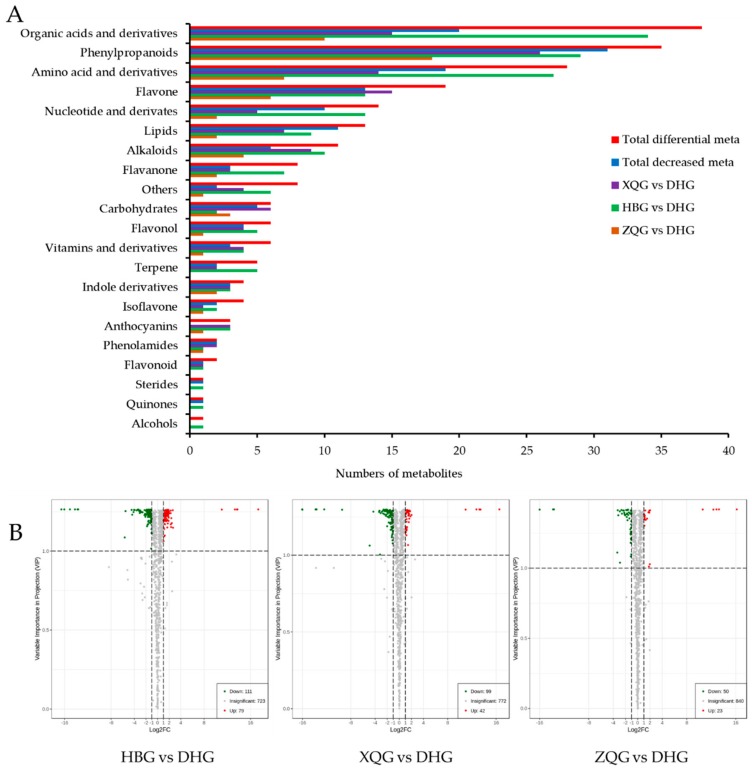
Number of different types of differential metabolites (**A**) and volcano plots of differential metabolites (**B**) of the tree comparison groups.

The highly dimensional and large metabolomics dataset required the application of univariate statistical analysis, PCA, and OPLS-DA methods to analyze all data taking into account the data characteristics, to accurately determine the differential metabolites. The univariate analysis (UVA) focuses largely on independent changes in metabolite levels and includes parametric tests and nonparametric tests. Based on the OPLS-DA results, the VIP can be used for preliminarily screening different metabolites between the treatment groups and control group. Moreover, differential metabolites can be further screened by combining the VIP value with the p-value or fold change value from univariate analysis. In this study, a total of 215 (HBG, 177; XQG, 124; and ZQG, 62; Figure 3B) differential metabolites were identified between the treatment groups and the control group (*p* < 0.05, VIP > 1). In total, all differential metabolites for all comparison groups were classified into 21 different categories (Figure 5A). Among all these differential metabolites, there were 38 organic acids and derivatives, 35 phenylpropanoids, 28 amino acids and derivatives, 19 flavone, 14 nucleotides and derivatives, 13 lipids, 11 alkaloids, 8 flavanone, 6 carbohydrates, 6 flavonols, 6 vitamins and derivatives, 5 terpenes, 4 indole derivatives, 4 isoflavones, 3 anthocyanins, 2 phenolamides, 2 flavonoids, 1 alcohol, 1 quinone, 1 steride, and 8 others (Figure 5A, Table S2). The major decreased categories were phenylpropanoids, organic acids and derivatives, amino acids and derivatives, and flavones. The differential metabolites of each comparison group were visualized with volcano plots (Figure 5B). In the plots, each point presents a metabolite; the abscissa indicates the fold-change (logarithm of base 2) of each substance in the comparison groups, and the ordinate indicates the VIP. There were 68 increased and 109 decreased metabolites in the HBG group versus DHG, 36 increased and 88 decreased metabolites in the XQG group versus DHG, and 21 increased and 41 decreased metabolites in the ZQG group versus DHG.

### 2.5. KEGG Annotation and Metabolic Pathway Analysis of Differential Metabolites for the Three Treatment Groups (HBG, XQG, and ZQG) versus the Control Group

The Kyoto Encyclopedia of Genes and Genomes (KEGG) database is the main public database of metabolic pathways and is used for the research of genes, expression information, and metabolite content in a general network. In the present study, we enriched the differential metabolites and classified them regarding pathways of the corresponding species ‘*Citrus sinensis*’ in which they were involved (Table S3). The detailed KEGG pathway diagrams for the three treatment groups (HBG, XQG, and ZQG) versus the control group are shown in Figure S4. Although the highly significant enrichment metabolic pathways (*p*-value < 0.01) were different in the three comparison groups, the biosynthesis of phenolic acids, namely, the “phenylpropanoid biosynthesis” pathway, was included in the highly significant enrichment metabolic pathways in all comparison groups (Figure S4, Table S3). The differential metabolites were classified into corresponding pathways according to the information of the pathway database (Table S3). The pathway analysis of the differential metabolites for the three treatment groups versus the control groups involved a total of 171 pathways. The major pathways are presented in bubble plots in Figure S5.

In the bubble plots, the rich factors refers to the ratio of the number of differential metabolites to the total number of metabolites detected in the corresponding pathway. The higher value represents the higher enrichment degree; the closer the *p*-value is to 0, the more significant the represented enrichment. The size of the bubble represents the number of metabolites enriched in the corresponding pathway. Thus, the pathways are annotated as whether they are considered important according to the analysis of enrichment and topology in Figure S5. On the basis of metabolic pathway analysis, the results indicated that the top five metabolic pathways, ranked in terms of the *p*-value, were “mineral absorption”, “phenylpropanoid biosynthesis”, “biosynthesis of alkaloids derived from the shikimate pathway”, “2-oxocarboxylic acid metabolism”, and “protein digestion and absorption” in HBG versus DHG; in XQG versus DHG, “glycosyl phosphatidylinositol (GPI)-anchor biosynthesis”, “autophagy-animal”, “pathogenic Escherichia coli infection”, “phenylpropanoid biosynthesis”, and “glycerophospholipid metabolism”; in ZQG versus DHG, “phenylpropanoid biosynthesis”, “glycerophospholipid metabolism”, “glycosylphosphatidylinositol (GPI)-anchor biosynthesis”, “autophagy-animal”, and “glycerophospholipid metabolism”. Among these metabolic pathways, all show *p*-values < 0.05 except for “glycosylphosphatidylinositol (GPI)-anchor biosynthesis” and “autophagy-animal” in the enrichment analysis of ZQG versus DHG comparison group (Figure S5, Table S3). It should be noted that phenylpropanoid biosynthesis is significant in all comparison groups. Phenolic acids and flavonoids encompass a large portion of the total metabolites in all three comparison groups. The total number of phenolic acids and flavonoids was 60 in HBG versus DHG, 53 in XQG versus DHG and 29 in ZQG versus DHG (Table S2).

### 2.6. Hierarchical Cluster Analysis of Differential Metabolites for the Three Treatment Groups (HBG, XQG, and ZQG) versus the Control Group

Normalization and hierarchical cluster analysis are often used to observe the variation rules of the significant differential metabolites. The significant differential metabolites in the three comparison groups showed many biologically similar or complementary functions, and the decreased or increased metabolites of the same metabolic pathways showed similar or opposite accumulation characteristics in the three comparison groups. According to the hierarchical cluster analysis, the differential metabolites were clustered using a complete-linkage method and displayed in thermograms. The total 215 differential metabolites are shown in Figure S6. In the thermograms, the color sequence range from orange to blue represents metabolites in the order of decreasing content. The hierarchical cluster analysis of the differential metabolites of the four types of fruit showed clear grouping pattern (Figure S6). The relative metabolite contents represented in the heatmaps are listed in Table S4.

### 2.7. Boxplots of Differential Metabolites for the Three Treatment Groups (HBG, XQG, and ZQG) versus the Control Group

**Figure 6:**
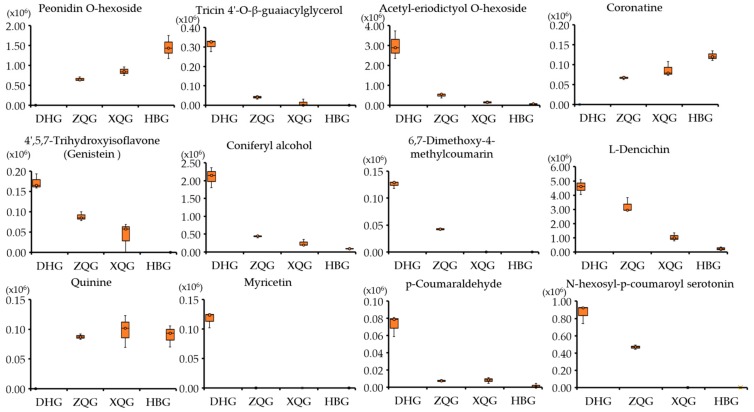
Boxplot of alkaloids, anthocyanins, phenylpropanoids, flavonoids, and other metabolites for the tree comparison groups. alkaloids: Quinine, l-Dencichin; Phenylpropanoids: Coniferyl alcohol, 6,7-Dimethoxy-4-methylcoumarin, *p*-Coumaraldehyde; Anthocyanins: peonidin *O*-hexoside; flavonoids: tricin 4′-*O*-β-guaiacylglycerol, acetyl-eriodictyol *O*-hexoside, myricetin, genistein (4′,5,7-Trihydroxyisoflavone); other metabolites: *N*-hexosyl-*p*-coumaroyl serotonin and Coronatine.

Boxplots were applied to display the variations in relative contents of the differential metabolites in each group. The relative contents of the differential metabolites between the three treatment groups (HBG, XQG, and ZQG) and the control group are listed in Table S5. Boxplots of alkaloids, anthocyanins, phenylpropanoids, and flavonoids (flavones, flavonols, and isoflavones) with relative contents that were significantly different for the three treatment groups (HBG, XQG, and ZQG) versus the control group are shown in Figure 6. There were one alkaloid, quinine and one other metabolite, coronatine, which both were significantly higher, and one alkaloid, L-dencichin, and one other metabolite, N-hexosyl-p-coumaroyl serotonin, which were significantly lower than those in the control group; one anthocyanin, peonidin *O*-hexoside, had a significantly higher content in the impacted fruit than in the control fruit; the contents of three phenylpropanoids, coniferyl alcohol, 6,7-dimethoxy-4-methylcoumarin, and p-coumaraldehyde, were significantly lower in the impacted fruits than in the control fruits; additionally, the significantly different flavonoids included tricin 4′-*O*-β-guaiacylglycerol, acetyl-eriodictyol *O*-hexoside, myricetin, and genistein (4′,5,7-trihy- droxyisoflavone) (*p*< 0.05, Figure 6). It is worth mentioning that genistein (4′,5,7- trihydroxyisoflavone) and *p*-coumaraldehyde in HBG versus DHG, *N*-hexosyl-*p*-coumaroyl serotonin, tricin 4′-*O*-β-guaiacylglycerol, and 6,7-dimethoxy-4- methylcoumarin in HBG versus DHG and in XQG versus DHG and myricetin in all comparison groups were barely detectable (*p* < 0.05) (Figure 6).

## 3. Discussion

### 3.1. Metabolites Identified in the Four Types of Fruit Peels

UHPLC-QQQ-MS-based metabolomics approach has been used to analyze the metabolites of peels of different colored mandarin fruits on one tree infected with HLB (Table S1). In previous studies, the metabolites of citrus fruits peels included neoeriocitrin, naringin, hesperidin, sinensetin, nobiletin, heptamethoxyflavone, tangeretin, 5-demethylnobiletin, tetramethyl-*O*-scutellarein, tetramethyl-*O*-isoscutellarein, pentamethoxyflavone, coumarins, neohesperidin, diosmin, poncirin, apigenin, rutin, quercetin, gallic acid, chlorogenic acids, p-coumaric acid, ferulic acid, caffeic acid, catechins, epicatechins, and kaempferol, most of which were classified as flavonoids or acids [19,20,21,22,23,24,25]. In the present study, 143 flavones, 85 phenylpropanoids, 36 flavonols, 28 flavonoids, 25 flavanones, 13 isoflavones, 11 anthocyanins, and 2 proanthocyanidins, including tricin, genistein, quercetin, 6,7-dimethoxy-4-methylcoumarin, and myricetin. Myricetin has many bioactivities such as antioxidant and antifungal properties [26,27]. Quercetin is an important flavonoid in citrus [28]. Moreover, 117 organic acids and derivatives, 98 amino acids and derivatives, 40 alkaloids, and 27 terpenes were also identified (Table S1), including morroniside, l-dencichin and 3-*O*-*p*-coumaroyl quinic acid. The 913 metabolites identified in all samples have been verified with respect to previously identified and provide reference values for the isolation and identification of functional compounds of fruit peels.

### 3.2. Differential Metabolites between the Three Treatment Groups (HBG, XQG, and ZQG) and the Control Group (DHG)

In this study, a total of 215 (HBG, 177; XQG, 124; and ZQG, 62) differential metabolites were identified in the comparison groups, including 109, 88, and 41 decreased and 68, 36, and 21 increased metabolites in the HBG versus DHG group, XQG versus DHG group, and ZQG versus DHG group, respectively (Figure 3B, Table S2). The major decreased categories were phenylpropanoids, organic acids and derivatives, amino acids and derivatives, and flavones. Metabolic pathway analysis of these differential metabolites revealed that the “phenylpropanoid biosynthesis” metabolic pathway had a *p*-value < 0.05 for enrichment analysis in all comparison groups, indicating that the relative content of phenylpropanoids was significantly different in all comparison groups (Figure S5, Table S3). Previous studies have shown that Huanglongbing (HLB) can significantly affected some secondary metabolism such as phenylpropanoid, flavonoid, and terpenoid pathways and primary metabolism such as fatty acid, pentose phosphate, and glycerolipid pathways [29]. The hierarchical cluster analysis of the differential metabolites suggested that those in the HBG, XQG, and ZQG groups and the DHG group showed distinct grouping patterns (Figure S6, Table S4). The results indicate that metabolites with the same characteristics were often biologically similar or complementary to each other regarding their functions or were negatively or positively regulated by the same metabolic pathway. The relative contents of three phenylpropanoids, four alkaloids, two flavones and one anthocyanin, flavonol, and isoflavone were significantly different between the three treatment groups (HBG, XQG, and ZQG) and the control group (DHG) (Figure 6). The phenylpropanoid content varied greatly among the flavedo (outer colored part of the peel) of the different groups [30], and the change in phenylpropanoid content was accompanied by changes in color and antioxidant activity [22,31,32]. Alkaloids were also the main component of citrus peels and have bioactive functions, such as antifungal and antioxidant activity [33]. The phenylpropanoids, flavones, anthocyanins, flavonols, and isoflavone identified in this study also belong to the flavonoid classification group [25]. It has been found that the flavonoids, antifungal activities, and antioxidant activity were significantly lower in the treatment groups than in the control group [34]. Therefore, future studies should focus on phenylpropanoid pathways and clarify which metabolites play vital roles in the pigmentation of mandarin citrus fruit peels.

## 4. Plant Materials and Treatments

### 4.1. Samples

The *Citrus reticulata* cv. ‘Shatangju’ was cultivated in Guangzhou City (23°9′ N, 113°9′ W) in Guangdong Province, China. The sampled citrus trees were managed in the same way as those in this citrus orchard. All trees were infected by *Candidatus* Liberibacter sp. There were significant differences in color of fruits during the maturation period. The fruits on the same tree were classified into four types according to the color: whole green fruits (HBG), top-yellow and base-green fruits (XQG), whole light-yellow fruits (ZQG), and whole dark yellow fruits (DHG). All fruits used for the metabolome analysis were collected on 5 January 2019. Three biological replicates were collected per sample, each with 60 fruits randomly collected from 10 ‘Shatangju’ trees in the same plot of the research orchard. The fruits were transported back to the laboratory, and the peels were carefully excised with scalpels, collected, frozen in liquid nitrogen, roughly ground, and kept at −80 °C for further research.

### 4.2. Extraction Process for Metabolite Analysis

Samples of the fruit peels were ground to a fine powder in liquid nitrogen and were thoroughly mixed. Then, a ca. 3 g sample was freeze-dried and crushed using a mixer mill (MM 400, Retsch, Haan, Germany) with a zirconia bead for 1.5 min at 30 Hz. A total of 100 mg of each powdered sample was dissolved in 1.0 mL of extracting solution (70% aqueous methanol) and extracted overnight at 4 ℃ on a rotating wheel, during which the samples were vortexed three times to ensure complete extraction. After extraction, the mixtures were centrifuged at 10,000× *g* for 10 min, and the supernatants were isolated (CNWBOND Carbon-GCB SPE Cartridge, 250 mg, 3 mL ANPEL, Shanghai, China) and filtered (SCAA-104, 0.22 μm pore size; ANPEL, Shanghai, China) before LC-MS analysis. In addition, a quality-control sample (mix) was prepared by mixing an equal amount of all samples. A quality control sample was run every 10 injections to monitor the stability of the analytical conditions during the assay.

### 4.3. HPLC and ESI-Q TRAP-MS/MS Conditions

#### 4.3.1. HPLC Separation Conditions

The peel extracts were analyzed using an LC-ESI-MS/MS system (HPLC, Shim-pack UFLC SHIMADZU CBM30A system, Kyoto, Japan; MS, Applied Biosystems 6500 Q TRAP, Foster City, CA, USA) equipped with a C18 column (Waters ACQUITY UPLC HSS T3, 1.8 μm, 2.1 mm × 100 mm). The solvent system was ultra-pure water containing 0.04% acetic acid as mobile phase A and acetonitrile containing 0.04% acetic acid as mobile phase B. The A:B (*v*:*v*) gradient program was as follows: 95:5 (*v*:*v*) at 0 min, 5:95 (*v*:*v*) at 11.0 min, 5:95 (*v*:*v*) at 12.0 min, 95:5 (*v*:*v*) at 12.1 min, and 95:5 (*v*:*v*) at 15.0 min. The flow rate was held at 0.40 mL/min. The column temperature was maintained at 40 °C. The injection volume of peel extract was 2 μL. The effluent was connected to an ESI-triple quadrupole-linear ion trap (Q TRAP)-MS [35].

#### 4.3.2. ESI-Q TRAP-MS/MS

Linear ion trap (LIT) and triple quadrupole (QQQ) scans were acquired on a triple quadrupole-linear ion trap mass spectrometer (Q TRAP, Applied Biosystems API 6500 Q TRAP LC/MS/MS System, Foster City, CA, USA) equipped with an ESI Turbo Ion-Spray interface operating in positive ion mode and controlled by Analyst 1.6.3 software (AB Sciex, Framingham, MA, USA). The ESI source operation parameters were as follows: the source temperature was 500 °C; the ion spray voltage (IS) was 5.5 kV; the ion source gas I (GSI), gas II (GSII), and curtain gas (CUR) were set at 55, 60, and 25 psi, respectively; the collision gas (CAD) was set to high. Instrument tuning and mass calibration were performed with 10 and 100 μmol/L polypropylene glycol solutions in QQQ and LIT modes, respectively. QQQ scans were acquired as MRM experiments with the collision gas (nitrogen) set to 5 psi. The DP and CE for individual MRM transitions was performed with further DP and CE optimization. A specific set of MRM transitions was monitored for each period according to the metabolites eluted within this period [35].

### 4.4. Qualitative and Quantitative Determination of Metabolites

#### 4.4.1. Qualitative Determination of Metabolites

The qualitative analysis of the primary and secondary mass spectrometry data were performed based on the self-built database MWDB (Metware Biotechnology Co., Ltd. Wuhan, China) and the public database of metabolite information and also based on second order spectrum. The interference from isotope signals, repetitive signals of K^+^, Na^+^, and NH_4_^+^ ions and fragment ions derived from other larger molecules were removed when qualitative analysis of the metabolites was performed. Metabolite structure analysis was conducted in reference to the existing public databases of mass spectrometry, such as Mass Bank (http://www.massbank.jp), KNAPSAcK (http://kanaya.naist.jp/KNApSAcK) and METLIN (http://metlin.scripps.edu/index.php).

#### 4.4.2. Quantitative Determination of Metabolites

Metabolite quantification was accomplished with data acquired in multiple reaction monitoring (MRM) mode of QQQ mass spectrometry. In MRM mode, the quadrupole first screened for precursor ions of target substances while screening out any ions derived from substances of different molecular weights to preliminarily eliminate their interference. The precursor ions were fragmented by induced ionization in the collision chamber to form several fragment ions. Then, unique fragment ions were selected with desired characteristics QQQ and to eliminate the interference from nontarget ions. This step aims to make the quantification more accurate and improve the repeatability. After metabolite mass spectrometry data were obtained for different samples, all mass spectrum peaks were subjected to area integration. The mass spectrum peaks of the same metabolite in different samples were integral-corrected. According to the RT and peak shape of each metabolite, the mass spectrum peak of each metabolite in each sample was corrected to screen out differential metabolites between each comparison group, thereby ensuring the accuracy of the qualitative and quantitative analyses [36].

### 4.5. Raw Data Preprocessing

In the present study, Analyst 1.6.3 software (AB Sciex, Framingham, MA, USA) was used to process mass spectrometry data. The IntelliQuan algorithm were used to integrated peak areas [35,37], and a series of data management steps was performed on the raw data to promote data analysis. Firstly, single mass spectrum peaks were filtered to retain peak area data for those peaks that contained null values accounting for no more than 50% of the whole data points, either in a single group or in all groups. secondly, missing values in the raw data were simulated via missing value recoding in which missing values were replaced with one half of the minimum values. Finally, the mass spectrum data of each sample was normalized according to the total ion current (TIC), and the metabolites were identified according to the MRM metabolite detection multipeak plot (ion flow spectra extracted from multiple analytes, XIC) [35,37].

### 4.6. Basis Data Analysis

Principal component analysis (PCA) of all samples, including QC samples, was performed to preliminarily understand the overall metabolic difference between the samples in each group and the variation between the samples in the group. Data were log-transformed and mean-centered using SIMCA v14.1 software (MKS Data Analytics Solutions, Umea, Sweden) and followed by automated modelling analysis to perform principal component analysis (PCA) [38,39].

For better visualization and further analysis of the data, orthogonal projections to latent structures-discriminant analysis (OPLS-DA) were performed for data evaluation. Data were log-transformed and subjected to unit variance scaling by SIMCA v14.1 software. The first principal component (PC1) was subjected to OPLS-DA modeling. *R*^2^*Y* and *Q*^2^ were used to evaluate the validity of the model. Permutation tests were performed multiple times (*n* = 200) to generate different random *Q*^2^ values that were used to further test the model validity [40]. The OPLS-DA was applied to filter out orthogonal metabolite variables that were not related to categorical variables, allowing the analysis of nonorthogonal variables and orthogonal variables separately to obtain more reliable information about metabolite differences between the experimental groups and the information about group-group correlations.

All metabolites identified in all samples were analyzed by ANOVA Univariate Analyses (UVA), i.e., Student’s t-test and analysis of variance were used to compare the relative contents of metabolites of each comparison group [41]. In the present study, two screening criteria, *p*-value < 0.05 and variable importance in the projection (VIP) of PC1 in the OPLS-DA model of ≥1 were used for screening differential metabolites. The Venn plots and boxplots were carried out by using R (http://www.r-project.org/).

### 4.7. Kyoto Encyclopedia of Genes and Genomes (KEGG) Annotation and Metabolic Pathway Analysis of Differential Metabolites

The KEGG Pathway database (https://www.kegg.jp/kegg/pathway.html) not only provides all possible metabolic pathways but also provides a comprehensive description of the enzymes that catalyze each step of a metabolic reaction [41]. The rich factors and *p*-value were used for enrichment analysis and topological analysis of the pathways. The rich factors were the ratios between the number of differentially accumulated metabolites in the corresponding pathway and the total number of metabolites detected in the detection nodes of the pathway. The larger the value, the greater the enrichment degree. The closer the *p*-value was to 0, the more significant the enrichment [42].

## 5. Conclusions

In the present study, a UHPLC-QQQ-MS-based metabolomics approach was implemented to systematically evaluate the difference in metabolites between fruit peels in three treatment groups (HBG, XQG, and ZQG) and those in a control group. A total of 913 metabolites were detected, 883 of which were shared by all samples. A total of 47 differential metabolites were common in the three comparison groups. Phenylpropanoids, organic acids and derivatives, amino acids and derivatives, and flavones were most decreased. At the same time, 2 unique metabolites, *O*-caffeoyl maltotriose and myricetin, were detected only in DHG samples. *O*-caffeoyl maltotriose and myricetin could be used as a marker to distinguish from the other mandarin fruits infected with HLB in early stage. The “phenylpropanoid biosynthesis” pathway was significantly enriched. In summary, the present work substantially contributes to the knowledge of metabolite compositions and functional compounds regarding the pigmentation and antifungal activity of mandarin fruit peels. The decreased metabolites and “phenylpropanoid biosynthesis” pathway will have considerable potential for further researching the influencing mechanism of Huanglongbing for citrus fruits development.

## Supplementary Materials

The following are available online, Table S1: A list of the 913 metabolites detected in this study; Table S2: The differential metabolites of HBG/XQG/ZQG versus DHG, respectively; Table S3: KEGG annotated results of differential metabolites of HBG/XQG/ZQG versus DHG; Table S4: The relative metabolite contents represented in the heatmaps of HBG/XQG/ZQG versus DHG; Table S5: The relative contents of differential metabolites between the three treatment groups (HBG, XQG, and ZQG) and the control group (DHG); Supplementary Figures: Figure S1. Phenotypic appearance of four types of mandarin fruits *Citrus reticulata* “Shatangju”; Figure S2. Total ions current overlaps of the three quality control samples by mass spectrometry detection; Figure S3. Principal component analysis (PCA) and orthogonal projections to latent structures-discriminant analysis (OPLS-DA) results; Figure S4. Metabolic pathways with different color dots representing the differential compounds for the tree comparison groups; Figure S5. Pathway analysis of differential metabolites for the tree comparison groups; Figure S6. Heatmap of hierarchical clustering analysis of differential metabolites for the tree comparison groups and editing certificate.
