# Supplementary material for: Metabolomics Analysis of the Peels of Different Colored Citrus Fruits (Citrus reticulata cv. ‘Shatangju’) During the Maturation Period Based on UHPLC-QQQ-MS
Source: Molecules. 2020 Jan 17;25(2):396. doi: 10.3390/molecules25020396 (PMC7024170; doi:10.3390/molecules25020396)

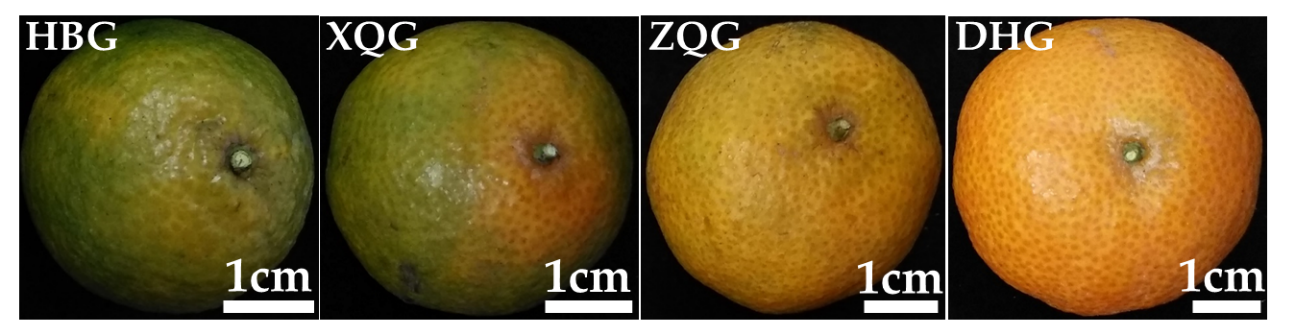


**Figure S1.** Phenotypic appearance of four types of mandarin fruits *Citrus reticulata* “Shatangju”.

HBG, whole green fruits; XQG, the top yellow and base green fruits; ZQG, the light-yellow fruits; DHG, the dark yellow fruit (control group).


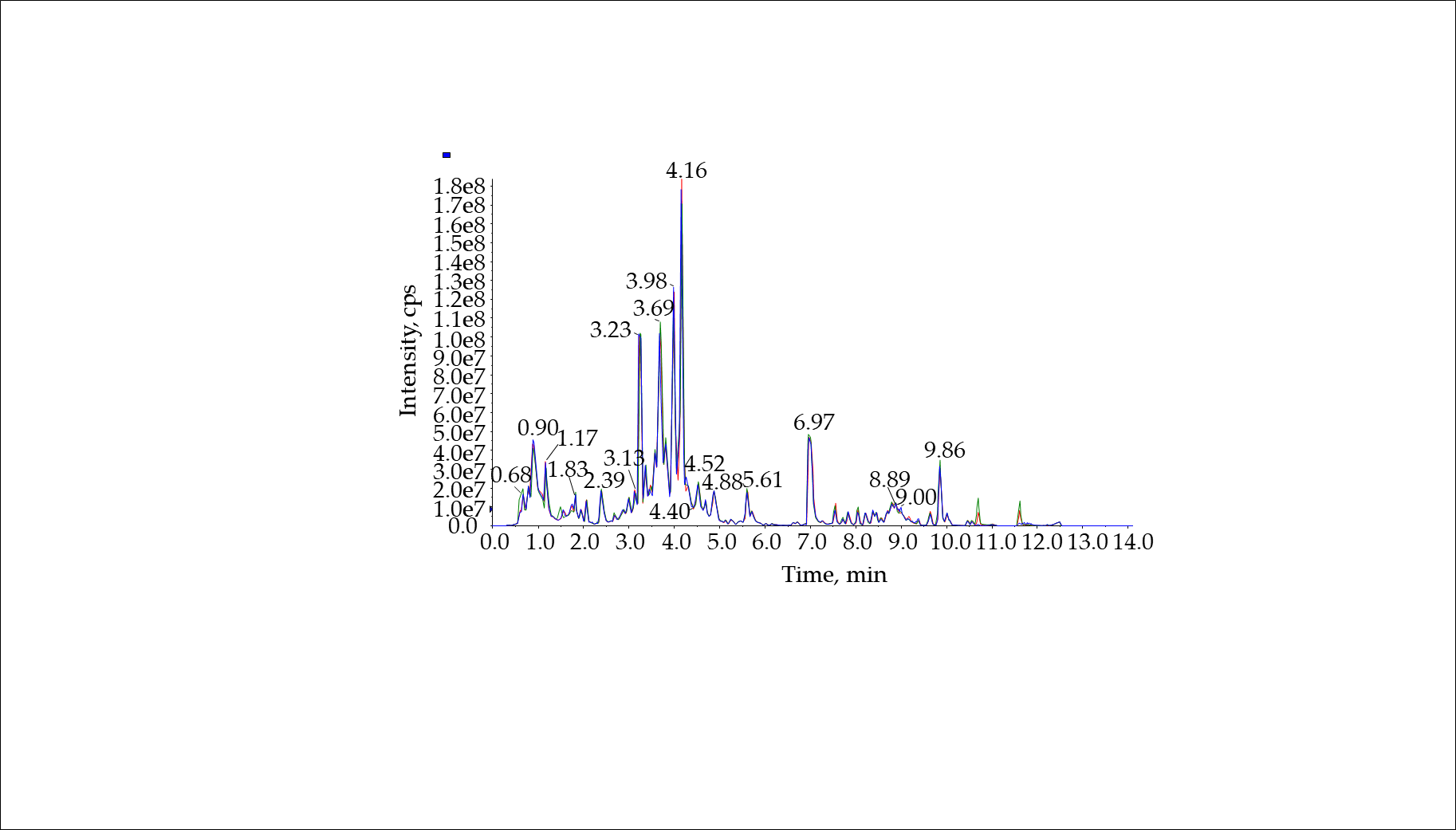


**Figure** **S2.** Total ions current overlaps of the three quality control samples by mass spectrometry detection.


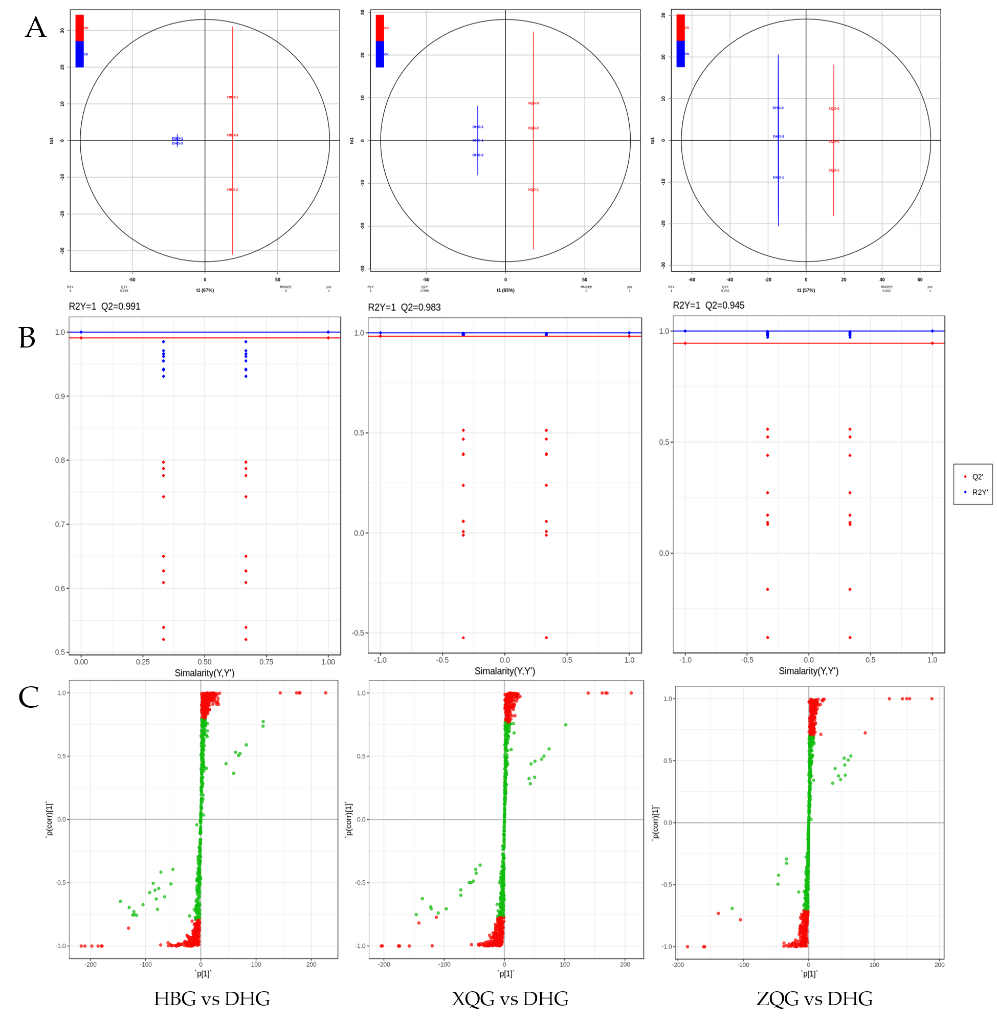


**Figure S3.** Principal component analysis (PCA) and orthogonal projections to latent structures-discriminant analysis (OPLS-DA) results.

(A) Score scatter plot of the OPLS-DA model for the tree comparison groups. (B) Permutation test of the OPLS-DA model for the tree comparison groups;(C) OPLS-DA S-plot for the tree comparison groups.

**Figure S4.** Metabolic pathways with different color dots representing the differential compounds for the tree comparison groups.

A:HBG versus DHG; B:XQG versus DHG; C:ZQG versus DHG. Differential metabolites are marked on the KEGG pathway diagram in bright green (indicating down-regulation) and bright red (indicating up-regulation).

HBG vs XQG

HBG vs ZQG

XQG vs ZQG


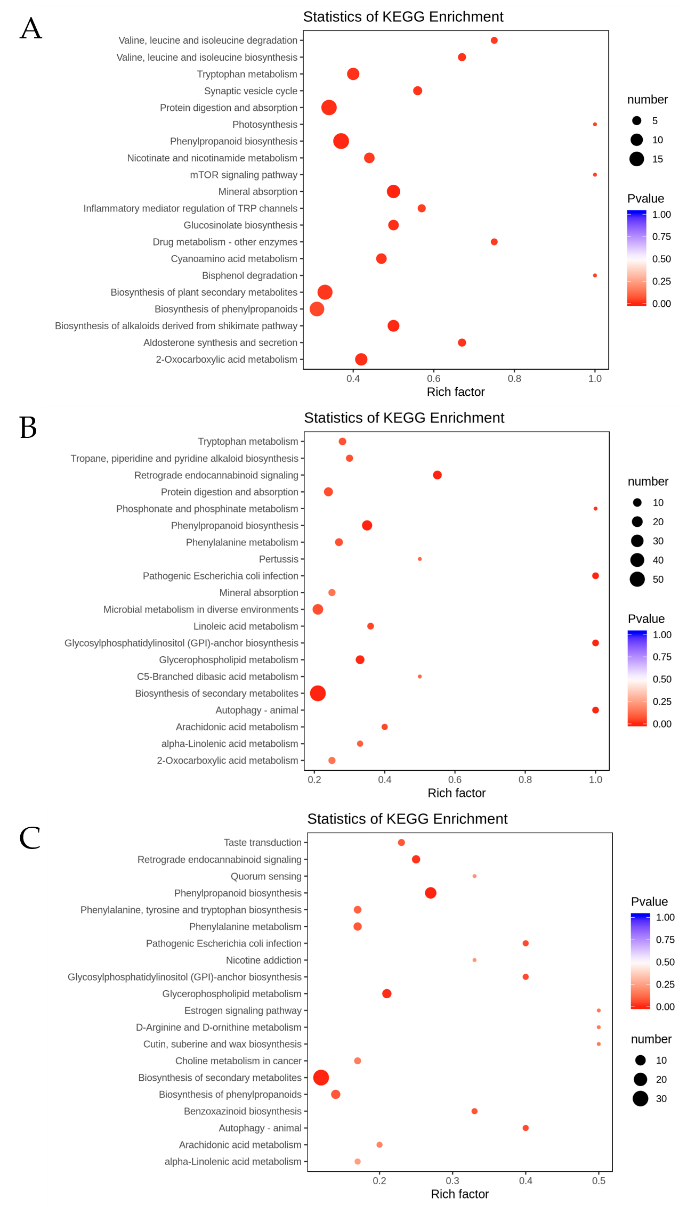


**Figure S5.** Pathway analysis of differential metabolites for the tree comparison groups.

A: HBG versus DHG; B: XQG versus DHG; C:ZQG versus DHG .

HBG vs XQG

HBG vs ZQG

XQG vs ZQG

Each bubble in the plot represents a metabolic pathway whose abscissa and bubble size indicate the magnitude of the rich factors and metabolites number of the pathway in the topological analysis, respectively. The bubble colors represent the P-values of the enrichment analysis, with darker colors showing a higher degree of enrichment.

**Figure S6.** Heatmap of hierarchical clustering analysis of differential metabolites for the tree comparison groups.

The abscissa indicates the four groups samples, and the ordinate indicates the differential metabolites of the tree comparison groups. The orange segments indicate relatively high content of metabolites, while the green segments indicate a relatively low content of metabolites. The relative metabolite contents represented by colour segments at the corresponding locations are listed in Table S4.


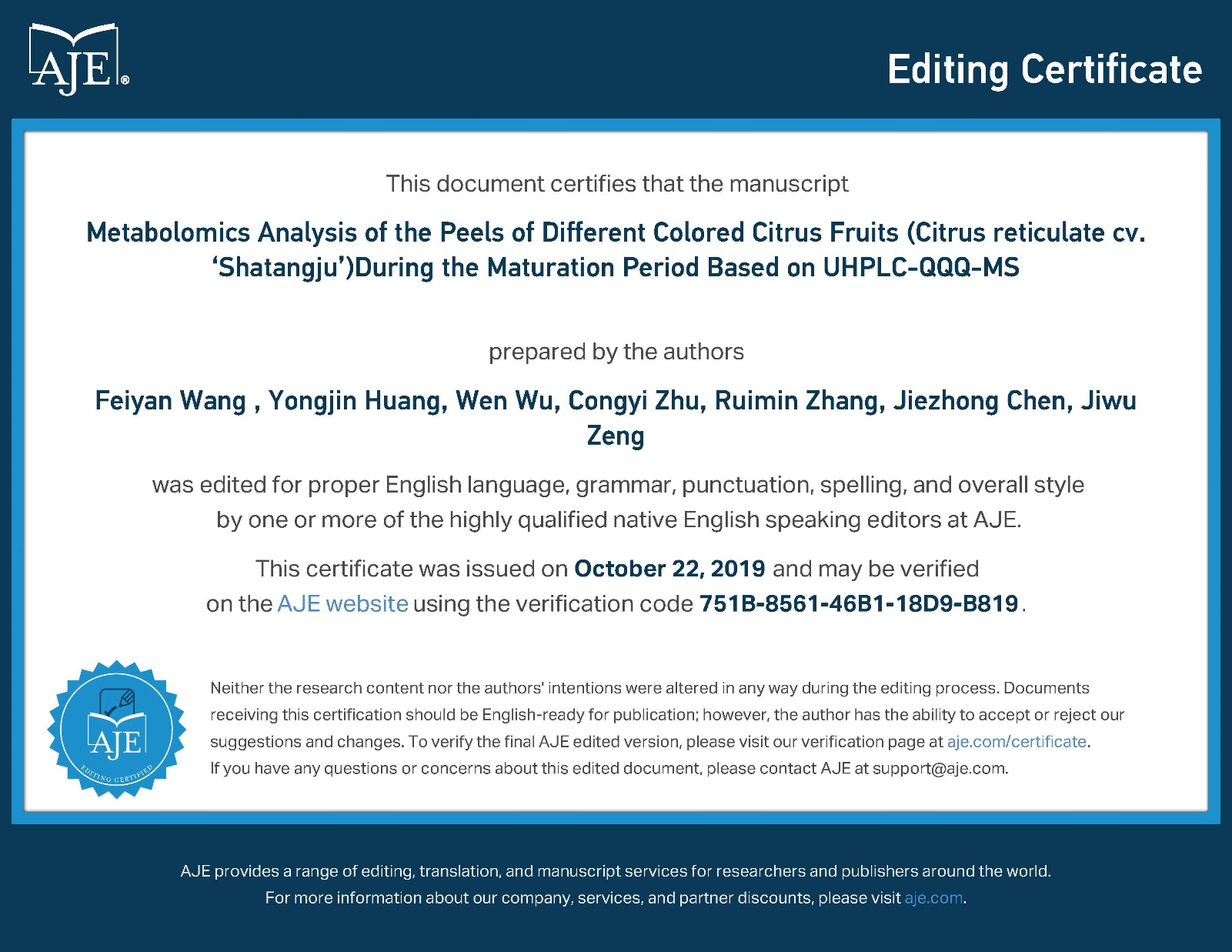

Supplement: Supplementary file 1 [file molecules-25-00396-s001.zip › molecules-664299-SI/Supplementary/Supplementary figure.docx]
