# Supplementary figures and images for: Metabolomics Analysis of the Peels of Different Colored Citrus Fruits (Citrus reticulata cv. ‘Shatangju’) During the Maturation Period Based on UHPLC-QQQ-MS
Source: Molecules. 2020 Jan 17;25(2):396. doi: 10.3390/molecules25020396 (PMC7024170; doi:10.3390/molecules25020396)

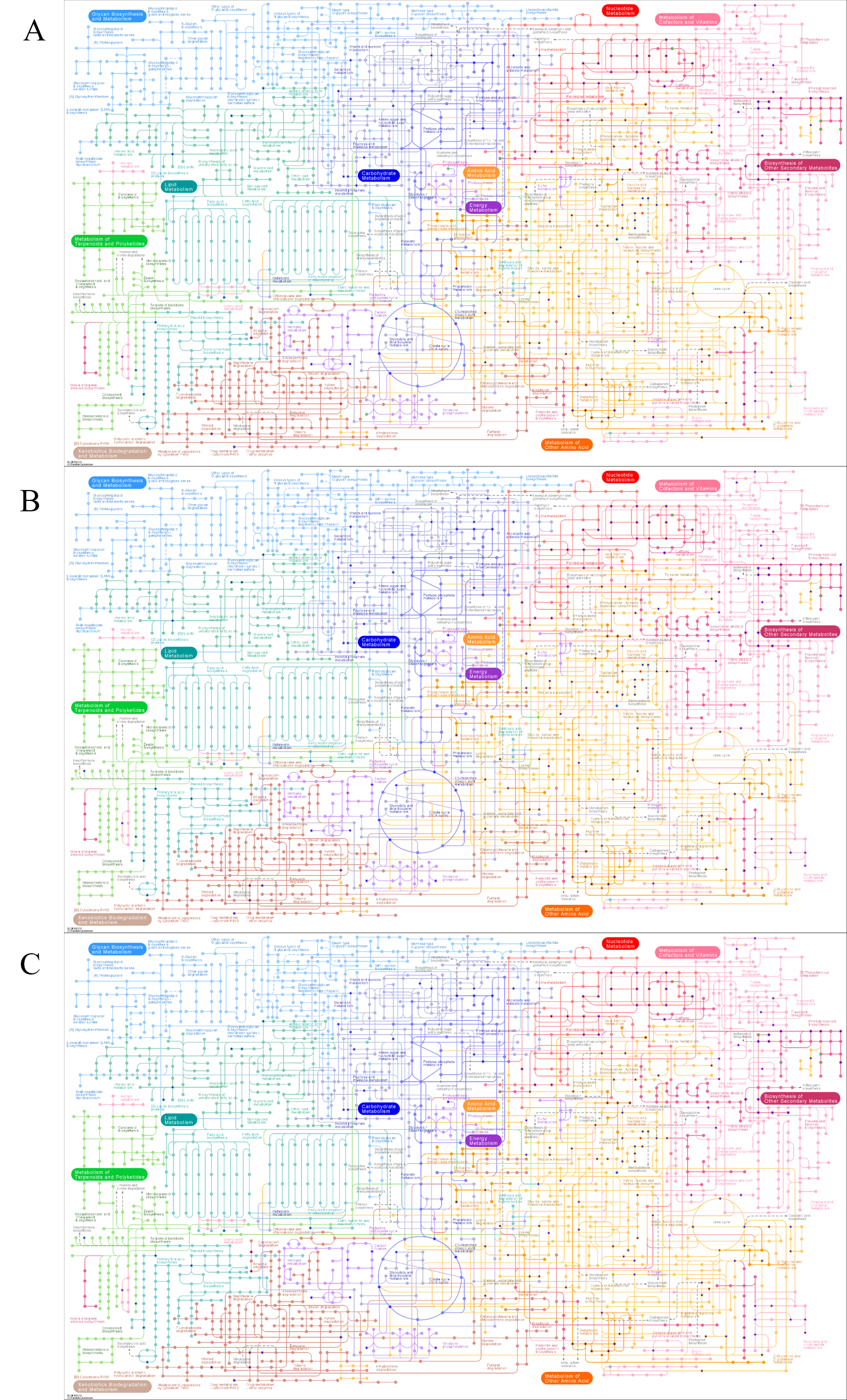

Supplement: Supplementary file 1 [file molecules-25-00396-s001.zip › molecules-664299-SI/Supplementary/Figure S4..tif]

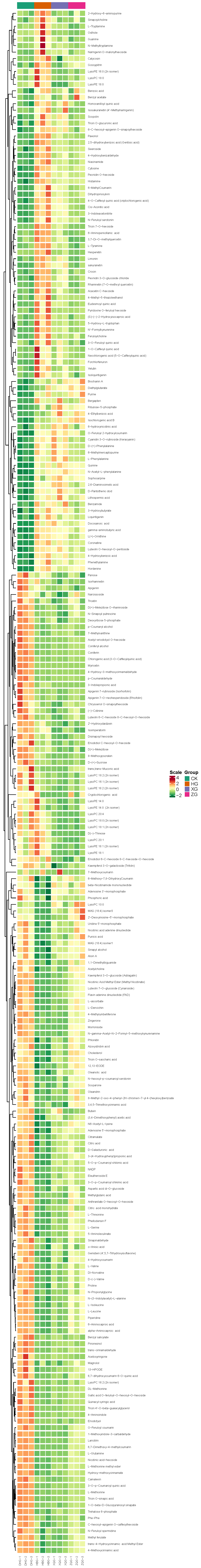

Supplement: Supplementary file 1 [file molecules-25-00396-s001.zip › molecules-664299-SI/Supplementary/Figure S6..tiff]
